# Supplementary material for: An H3K14ub-H3K9me3 feedback circuit governs heterochromatin spreading and inheritance in fission yeast
Source: Nat Commun. 2026 Mar 5;17:3483. doi: 10.1038/s41467-026-70276-8 (PMC13079825; doi:10.1038/s41467-026-70276-8)
Supplement: Supplementary file 1 — Supplementary Information [file 41467_2026_70276_MOESM1_ESM.pdf]

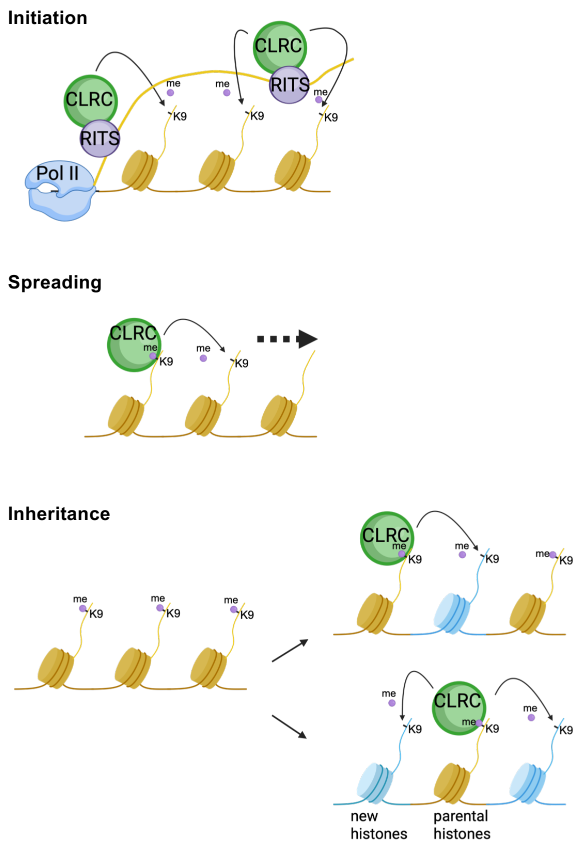

Figure S1. Schematic model of heterochromatin initiation, spreading, and inheritance. Created in BioRender. Jia, S. (2026) https://BioRender.com/94e5xb9.

Figure S2

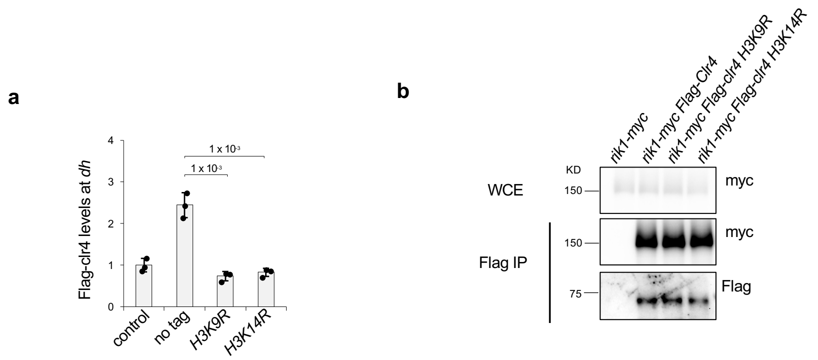

Figure S2. The effects of H3K9R and H3K14R on CLRC localization and complex integrity.

(a) ChIP analyses of Flag-Clr4 levels at *dh*. Data are presented as mean ± s.d. from 3 biological replicates. Statistical significance was assessed using two-tailed unpaired Student's *t*-tests for the indicated pairwise comparisons. Exact *P*-values are indicated above each comparison.

(b) Co-immunoprecipitation of Flag-Clr4 and Rik1-myc. Cell lysates from indicated strains were incubated with Flag-agarose beads, washed and eluted with SDS loading buffer. The samples were resolved by SDS-PAGE and western blot were performed with myc and Flag antibodies.

Figure S3

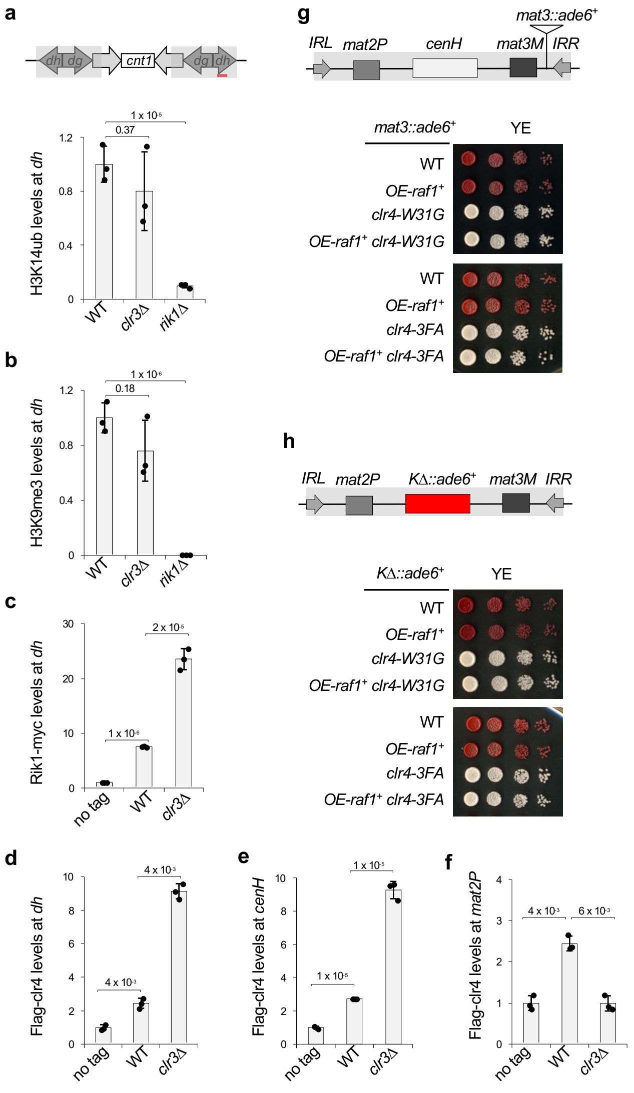

Figure S3. Clr3 regulates H3K14ub.

(a) Top, schematic diagram of the pericentric region of chromosome I. Red bar indicate the position of PCR fragments used in qPCR. Bottom, ChIP analyses of H3K14ub levels at *dh*. Data are presented as mean ± s.d. from 3 biological replicates.

(b) ChIP analyses of H3K9me3 levels at *dh*. Data are presented as mean ± s.d. from 3 biological replicates. Statistical significance was assessed using two-tailed unpaired Student's *t*-tests for the indicated pairwise comparisons. Exact *P*-values are indicated above each comparison.

(c) ChIP analyses of Rik1-myc levels at *dh*. Data are presented as mean ± s.d. from 3 biological replicates. Statistical significance was assessed using two-tailed unpaired Student's *t*-tests for the indicated pairwise comparisons. Exact *P*-values are indicated above each comparison.

(d,e,f) ChIP analyses of Flag-Clr4 levels at *dh*, *cenH*, and *mat2P*. Data are presented as mean ± s.d. from 3 biological replicates. Statistical significance was assessed using two-tailed unpaired Student's *t*-tests for the indicated pairwise comparisons. Exact *P*-values are indicated above each comparison.

(g) Serial dilution analysis of indicated strains to measure the expression of *mat3::ade6+*.

(h) Serial dilution analysis of indicated strains to measure the expression of *KΔ::ade6+*.

Figure S4

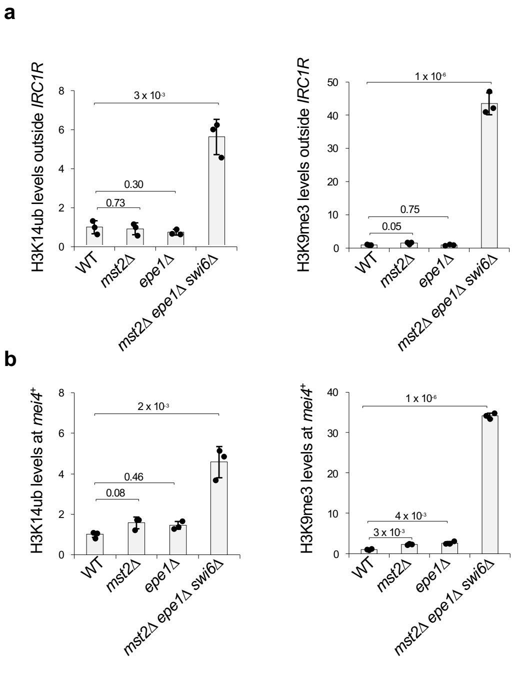

Figure S4. Mst2 and Epe1 synergistically limit heterochromatin expansion.

(a) ChIP analyses of H3K14ub and H3K9me3 levels at outside of *IRC1R* boundary. Data are presented as mean ± s.d. from 3 biological replicates. Statistical significance was assessed using two-tailed unpaired Student's *t*-tests for the indicated pairwise comparisons. Exact *P*-values are indicated above each comparison.

(b) ChIP analyses of H3K14ub and H3K9me3 levels at heterochromatin island *mei4*+*.* Data are presented as mean ± s.d. from 3 biological replicates. Statistical significance was assessed using two-tailed unpaired Student's *t*-tests for the indicated pairwise comparisons. Exact *P*-values are indicated above each comparison.
