# Supplementary material for: An H3K14ub-H3K9me3 feedback circuit governs heterochromatin spreading and inheritance in fission yeast
Source: Nat Commun. 2026 Mar 5;17:3483. doi: 10.1038/s41467-026-70276-8 (PMC13079825; doi:10.1038/s41467-026-70276-8)
Supplement: Supplementary file 2 — Description of Additional Supplementary Files [file 41467_2026_70276_MOESM2_ESM.pdf]

Descriptions of Additional Supplementary Files

Supplementary Data 1: Yeast strains used in this study.

Supplementary Data 2: DNA oligonucleotide sequences used in this study.
