# Supplementary material for: An H3K14ub-H3K9me3 feedback circuit governs heterochromatin spreading and inheritance in fission yeast
Source: Nat Commun. 2026 Mar 5;17:3483. doi: 10.1038/s41467-026-70276-8 (PMC13079825; doi:10.1038/s41467-026-70276-8)
Supplement: Supplementary file 4 — Reporting Summary [file 41467_2026_70276_MOESM4_ESM.pdf]

nature portfolio

Corresponding author(s): Songtao Jia
Last updated by author(s): Nov 9, 2025

Reporting Summary

Nature Portfolio wishes to improve the reproducibility of the work that we publish. This form provides structure for consistency and transparency in reporting. For further information on Nature Portfolio policies, see our Editorial Policies and the Editorial Policy Checklist.

Statistics

For all statistical analyses, confirm that the following items are present in the figure legend, table legend, main text, or Methods section.

n/a	Confirmed	
☐	☒	The exact sample size (*n*) for each experimental group/condition, given as a discrete number and unit of measurement
☒	☐	A statement on whether measurements were taken from distinct samples or whether the same sample was measured repeatedly
☐	☒	The statistical test(s) used AND whether they are one- or two-sided<br>*Only common tests should be described solely by name; describe more complex techniques in the Methods section.*
☒	☐	A description of all covariates tested
☒	☐	A description of any assumptions or corrections, such as tests of normality and adjustment for multiple comparisons
☐	☒	A full description of the statistical parameters including central tendency (e.g. means) or other basic estimates (e.g. regression coefficient) AND variation (e.g. standard deviation) or associated estimates of uncertainty (e.g. confidence intervals)
☐	☒	For null hypothesis testing, the test statistic (e.g. *F*, *t*, *r*) with confidence intervals, effect sizes, degrees of freedom and *P* value noted<br>*Give P values as exact values whenever suitable.*
☒	☐	For Bayesian analysis, information on the choice of priors and Markov chain Monte Carlo settings
☒	☐	For hierarchical and complex designs, identification of the appropriate level for tests and full reporting of outcomes
☒	☐	Estimates of effect sizes (e.g. Cohen's *d*, Pearson's *r*), indicating how they were calculated

Our web collection on statistics for biologists contains articles on many of the points above.

Software and code

Policy information about availability of computer code

Data collection	No software used.
Data analysis	Timmomatic (v0.38), BWA (v0.7.17), SAMtools (v1.9), Picard (v2.19.0), GATK3 (v3.6-6), bedtools (v2.27.1)

For manuscripts utilizing custom algorithms or software that are central to the research but not yet described in published literature, software must be made available to editors and reviewers. We strongly encourage code deposition in a community repository (e.g. GitHub). See the Nature Portfolio guidelines for submitting code & software for further information.

Data

Policy information about availability of data

All manuscripts must include a data availability statement. This statement should provide the following information, where applicable:

- Accession codes, unique identifiers, or web links for publicly available datasets
- A description of any restrictions on data availability
- For clinical datasets or third party data, please ensure that the statement adheres to our policy

ChIP-seq data is available in the NCBI database under accession number PRJNA1300844.

Research involving human participants, their data, or biological material

Policy information about studies with human participants or human data. See also policy information about sex, gender (identity/presentation), and sexual orientation and race, ethnicity and racism.

Reporting on sex and gender	Not relevant.
Reporting on race, ethnicity, or other socially relevant groupings	Not relevant.
Population characteristics	Not relevant.
Recruitment	Not relevant.
Ethics oversight	Not relevant.

Note that full information on the approval of the study protocol must also be provided in the manuscript.

Field-specific reporting

Please select the one below that is the best fit for your research. If you are not sure, read the appropriate sections before making your selection.

[x] Life sciences   [ ] Behavioural & social sciences   [ ] Ecological, evolutionary & environmental sciences

For a reference copy of the document with all sections, see nature.com/documents/nr-reporting-summary-flat.pdf

Life sciences study design

All studies must disclose on these points even when the disclosure is negative.

Sample size	Not relevant.
Data exclusions	Not relevant.
Replication	Not relevant.
Randomization	Not relevant.
Blinding	Not relevant.

Reporting for specific materials, systems and methods

We require information from authors about some types of materials, experimental systems and methods used in many studies. Here, indicate whether each material, system or method listed is relevant to your study. If you are not sure if a list item applies to your research, read the appropriate section before selecting a response.

Materials & experimental systems

n/a	Involved in the study	
[ ]	[x]	Antibodies
[ ]	[ ]	Eukaryotic cell lines
[ ]	[ ]	Palaeontology and archaeology
[ ]	[ ]	Animals and other organisms
[ ]	[ ]	Clinical data
[ ]	[ ]	Dual use research of concern
[ ]	[ ]	Plants

Methods

n/a	Involved in the study	
[ ]	[x]	ChIP-seq
[ ]	[ ]	Flow cytometry
[ ]	[ ]	MRI-based neuroimaging

Antibodies

Antibodies used	H3K14ub (custom made), H3K9me3 (Active Motif, 39161), Myc (Sigma, C3956), Flag (Genscript A00187)
Validation	H3K14ub antibody was validated by western with nculeosome containing differnet ubiquitination as controls, and ChIP analysis with rik1Δ as control.<br>Myc antibody was validated by ChIP with no tag strain as control.<br>H3K9me3 antibody was validated by ChIP with clr4Δ control.<br>Flag antibody was validated by western with no tagged strain as control.

Eukaryotic cell lines

Policy information about cell lines and Sex and Gender in Research

Cell line source(s)	Not relevant.
Authentication	Not relevant.
Mycoplasma contamination	Not relevant.
Commonly misidentified lines (See ICLAC register)	Not relevant.

Palaeontology and Archaeology

Specimen provenance	Not relevant.
Specimen deposition	Not relevant.
Dating methods	Not relevant.

☐ Tick this box to confirm that the raw and calibrated dates are available in the paper or in Supplementary Information.

Ethics oversight	Not relevant.

Note that full information on the approval of the study protocol must also be provided in the manuscript.

Animals and other research organisms

Policy information about studies involving animals; ARRIVE guidelines recommended for reporting animal research, and Sex and Gender in Research

Laboratory animals	No animals used.
Wild animals	No wild animals used.
Reporting on sex	Not relevant.
Field-collected samples	Not relevant.
Ethics oversight	Not relevant.

Note that full information on the approval of the study protocol must also be provided in the manuscript.

Clinical data

Policy information about clinical studies

All manuscripts should comply with the ICMJE guidelines for publication of clinical research and a completed CONSORT checklist must be included with all submissions.

Clinical trial registration	Not relevant.
Study protocol	Not relevant.
Data collection	Not relevant.
Outcomes	Not relevant.

Dual use research of concern

Policy information about dual use research of concern

Hazards

Could the accidental, deliberate or reckless misuse of agents or technologies generated in the work, or the application of information presented in the manuscript, pose a threat to:

No	Yes	
☒	☐	Public health
☒	☐	National security
☒	☐	Crops and/or livestock
☒	☐	Ecosystems
☒	☐	Any other significant area

Experiments of concern

Does the work involve any of these experiments of concern:

No	Yes	
☒	☐	Demonstrate how to render a vaccine ineffective
☒	☐	Confer resistance to therapeutically useful antibiotics or antiviral agents
☒	☐	Enhance the virulence of a pathogen or render a nonpathogen virulent
☒	☐	Increase transmissibility of a pathogen
☒	☐	Alter the host range of a pathogen
☒	☐	Enable evasion of diagnostic/detection modalities
☒	☐	Enable the weaponization of a biological agent or toxin
☒	☐	Any other potentially harmful combination of experiments and agents

Plants

Seed stocks	Not relevant.
Novel plant genotypes	Not relevant.
Authentication	Not relevant.

ChIP-seq

Data deposition

☒ Confirm that both raw and final processed data have been deposited in a public database such as GEO.

☐ Confirm that you have deposited or provided access to graph files (e.g. BED files) for the called peaks.

Data access links<br>*May remain private before publication.*	https://www.ncbi.nlm.nih.gov/bioproject/PRJNA1300844
Files in database submission	WT-H3K9me3.R1.fq.gz<br>WT-H3K9me3.R2.fq.gz<br>WT-H3K9me3-input.R1.fq.gz<br>WT-H3K9me3-input.R2.fq.gz<br>rik1-H3K9me3.R1.fq.gz<br>rik1-H3K9me3.R2.fq.gz<br>rik1-H3K9me3-input.R1.fq.gz<br>rik1-H3K9me3-input.R2.fq.gz<br>WT-H3K14ub-input.R1.fastq.gz<br>WT-H3K14ub-input.R2.fastq.gz<br>rik1-H3K14ub-input.R1.fastq.gz<br>rik1-H3K14ub-input.R2.fastq.gz<br>rik1-H3K14ub.R1.fastq.gz<br>rik1-H3K14ub.R2.fastq.gz<br>WT-H3K14ub.R1.fastq.gz<br>WT-H3K14ub.R2.fastq.gz

Genome browser session (e.g. UCSC) | No longer applicable.

Methodology

Replicates	No
Sequencing depth	*Describe the sequencing depth for each experiment, providing the total number of reads, uniquely mapped reads, length of reads and whether they were paired- or single-end.*
Antibodies	H3K14ub (custom made), H3K9me3 (Active Motif, 39161)
Peak calling parameters	No used.
Data quality	Controls that are expected to abolish the modifications are also performed to make sure that signals are abolished.
Software	BWA, samtools, Picard, Trimmomatic, bedtools, GATK3, MACS2

Flow Cytometry

Plots

Confirm that:

- [ ] The axis labels state the marker and fluorochrome used (e.g. CD4-FITC).
- [ ] The axis scales are clearly visible. Include numbers along axes only for bottom left plot of group (a 'group' is an analysis of identical markers).
- [ ] All plots are contour plots with outliers or pseudocolor plots.
- [ ] A numerical value for number of cells or percentage (with statistics) is provided.

Methodology

Sample preparation	Not relevant.
Instrument	Not relevant.
Software	Not relevant.
Cell population abundance	Not relevant.
Gating strategy	Not relevant.

- [ ] Tick this box to confirm that a figure exemplifying the gating strategy is provided in the Supplementary Information.

Magnetic resonance imaging

Experimental design

Design type	Not relevant.
Design specifications	Not relevant.
Behavioral performance measures	Not relevant.

Acquisition

Imaging type(s)	Not relevant.
Field strength	Not relevant.
Sequence & imaging parameters	Not relevant.
Area of acquisition	Not relevant.

Diffusion MRI ☐ Used ☒ Not used

Preprocessing

Preprocessing software	Not relevant.
Normalization	Not relevant.

Normalization template	Not relevant.
Noise and artifact removal	Not relevant.
Volume censoring	Not relevant.

Statistical modeling & inference

Model type and settings	Not relevant.
Effect(s) tested	Not relevant.

Specify type of analysis: ☐ Whole brain ☐ ROI-based ☐ Both

Statistic type for inference (See Eklund et al. 2016)	Not relevant.
Correction	Not relevant.

Models & analysis

n/a	Involved in the study
☐	☐ Functional and/or effective connectivity
☐	☐ Graph analysis
☐	☐ Multivariate modeling or predictive analysis

Functional and/or effective connectivity	Not relevant.
Graph analysis	Not relevant.
Multivariate modeling and predictive analysis	Not relevant.
